# Diverse Inflammatory Response After Cerebral Microbleeds Includes Coordinated Microglial Migration and Proliferation

**DOI:** 10.1161/STROKEAHA.117.020461

**Published:** 2018-05-29

**Authors:** Sung Ji Ahn, Josef Anrather, Nozomi Nishimura, Chris B. Schaffer

**Affiliations:** 1 From the Meinig School of Biomedical Engineering, Cornell University, Ithaca, NY (S.J.A., N.N., C.B.S.); 2 Feil Family Brain and Mind Research Institute, Weill Cornell Medical College, New York, NY (J.A.).

**Keywords:** animal models, intracranial hemorrhages, inflammation, lasers, leukocytes, microglia, optical imaging

## Abstract

**Background and Purpose—:**

Cerebral microbleeds are linked to cognitive decline, but it remains unclear how they impair neuronal function. Infarction is not typically observed near microbleeds, suggesting more subtle mechanisms, such as inflammation, may play a role. Because of their small size and largely asymptomatic nature, real-time detection and study of spontaneous cerebral microbleeds in humans and animal models are difficult.

**Methods—:**

We used in vivo 2-photon microscopy through a chronic cranial window in adult mice to follow the inflammatory response after a cortical microhemorrhage of ≈100 µm diameter, induced by rupturing a targeted cortical arteriole with a laser.

**Results—:**

The inflammatory response included the invasion of blood-borne leukocytes, the migration and proliferation of brain-resident microglia, and the activation of astrocytes. Nearly all inflammatory cells responding to the microhemorrhage were brain-resident microglia, but a small number of CX3CR1^+^ and CCR2^+^ macrophages, ultimately originating from the invasion of blood-borne monocytes, were also found near the lesion. We found a coordinated pattern of microglia migration and proliferation, where microglia within 200 µm of the microhemorrhage migrated toward the lesion over hours to days. In contrast, microglia proliferation was not observed until ≈40 hours after the lesion and occurred primarily in a shell-shaped region where the migration of microglia decreased their local density. These data suggest that local microglia density changes may trigger proliferation. Astrocytes activated in a similar region as microglia but delayed by a few days. By 2 weeks, this inflammatory response had largely resolved.

**Conclusions—:**

Although microhemorrhages are small in size, the brain responds to a single bleed with an inflammatory response that involves brain-resident and blood-derived cells, persists for weeks, and may impact the adjacent brain microenvironment.

## Abstract 2

Supplemental Digital Content is available in the text.

Cerebral microbleeds (CMBs) are a manifestation of cerebral small vessel disease and are currently a major topic in clinical cerebrovascular research.^1^ CMBs vary in size from tens of micrometers to several millimeters and occur at increasing frequency with age,^2^ hypertension,^3^ cerebral amyloid angiopathy,^4^ anticoagulant treatment,^5^ and with genetic conditions, such as cerebral autosomal dominant arteriopathy with subcortical infarcts and leukoencephalopathy.^1,6^ While typically thought of as acutely asymptomatic, several case reports document CMBs leading to transient neurological episodes that include symptoms, such as paresthesia, weakness, and dysphasia in patients with cerebral amyloid angiopathy (where this is termed amyloid spells)^7^ or with cerebral autosomal dominant arteriopathy with subcortical infarcts and leukoencephalopathy.^6^ Several large studies have linked increased incidence of CMBs to accelerated cognitive decline and to increased risk for dementia.^1,8,9^ The cellular mechanisms by which CMBs affect the health and function of cells in the brain microenvironment, however, remain poorly understood.

In recent work, we have used a laser-based approach to induce CMBs in the cortex of rodents^10^ to explore the impact of these lesions on neurons and surrounding cells. We found that microhemorrhages do not lead to nearby cell death or degeneration of dendritic arbors^11^ or to long-term loss of neural responsivity.^12^ We did observe an increase in the density of inflammatory cells near the lesion although the classes of cells involved and their origin was not determined.^11^ Consistent with this, the few postmortem studies of CMBs in the literature tend to report increased presence of inflammatory cells rather than significant tissue infarction.^13–15^ Here, we used a laser-induced microhemorrhage model and 2-photon excited fluorescence imaging to examine the inflammatory response after a microhemorrhage, identifying some of the cell types involved, their spatiotemporal dynamics, and the mechanisms leading to increased inflammatory cell density near the lesion.

## Methods

The data that support the findings of this study are available from the corresponding author on reasonable request. All animal experiments were conducted in strict accordance with the recommendations in the Guide for the Care and Use of Laboratory Animals published by National Institutes of Health, and all animal procedures were approved by the Cornell University Institutional Animal Care and Use Committee (protocol numbers 2009-0043 and 2015-0029). Detailed Materials and Methods are provided in the online-only Data Supplement.

**Figure 1.**
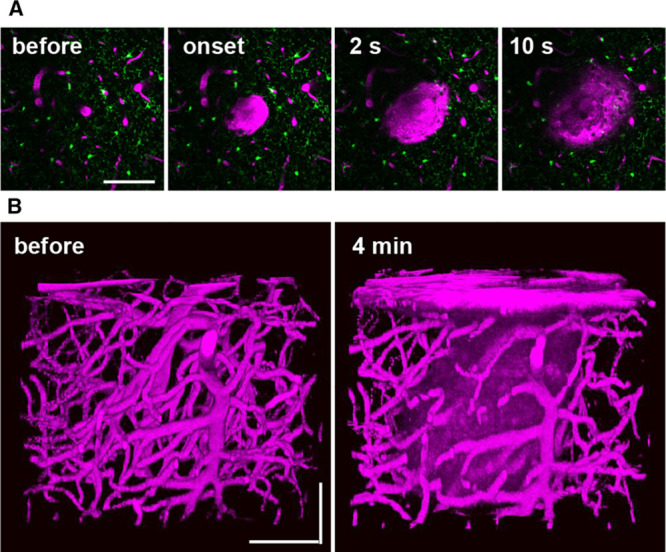
Femtosecond laser-induced cortical microhemorrhages. **A**, In vivo 2-photon excited fluorescence images of fluorescently-labeled blood plasma (magenta, intravenously injected Texas Red dextran) during creation of a microhemorrhage by ablation of a cortical penetrating arteriole. **B**, Three-dimensional reconstruction of the vasculature and extravagated plasma before and after the lesion. All scale bars are 100 µm.

To study the inflammatory response to a brain microhemorrhage, we implanted a chronic, glass-covered cranial window over the cortex of adult mice. To distinguish the response of different classes of inflammatory cells to a CMB, we used transgenic fluorescent reporter mouse lines and bone marrow transplant chimeras to label different classes of inflammatory cells. After 3 weeks of recovery, we used a laser-based approach to rupture targeted penetrating arterioles, vessels that branch from the surface arteriole network and dive into the brain to feed capillary beds.^12^ We irradiated the edge of the vessel lumen with short bursts of tightly-focused femtosecond laser pulses with sufficient energy to cause ionization of the material at the laser focus. When targeting ≈20 µm diameter penetrating arterioles, an ≈100 µm diameter CMB was reliably produced in the cortex^10–12^ (Figure 1; Movie I in the online-only Data Supplement), comparable to the size of the smaller bleeds found in postmortem human brain.^16^ This laser irradiation deposited relatively little total energy, so there was minimal collateral damage to surrounding tissue from the laser itself. The ruptured wall of the targeted vessel clotted within a few seconds and restricted the size of the hematoma.^11^ Blood flow in the targeted penetrating was not disrupted, so there is no ischemic injury in this model.^10^ This approach allows us to control the time and location of a CMB in the cortex, allowing the use of in vivo 2-photon excited fluorescence microscopy to follow the cellular response to the injury. We followed the invasion, migration, and proliferation of various classes of inflammatory cells at the lesion site over hours to weeks using 2-photon excited fluorescence imaging. We also quantified microglia proliferation and confirmed distance and time-dependent cell density changes after the lesion using histological approaches in animals that were euthanized at different time points after inducing a microhemorrhage. Finally, we developed phenomenological computational models of the microglia response to the microhemorrhage based on the migration and proliferation rates we observed.

## Results

**Figure 2.**
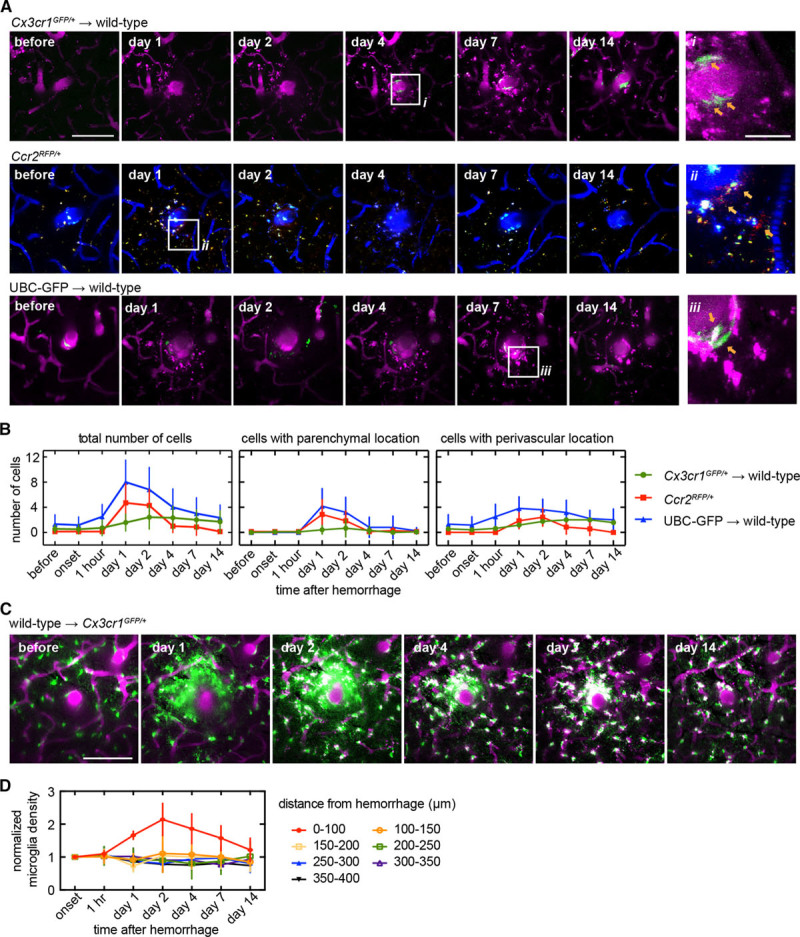
Invasion of a small number of blood-borne inflammatory cells and an increase in density of brain-resident microglia were observed over a few days near the microhemorrhage. **A**, Axial projections of 2-photon image stacks showing the response of different genetically-labeled inflammatory cell types for 2 wk after a microhemorrhage. In wild-type animals receiving a *Cx3cr1*^*GFP/+*^ bone marrow transplant (*Cx3cr1*^*GFP/+*^→wild-type), labeled cells are patrolling monocytes (**top**, green, GFP [green fluorescent protein]). In *CCR2*^*RFP/+*^ animals, labeled cells are inflammatory monocytes (**middle**, red, RFP [red fluorescent protein]; blue, intravenouslyinjected Cascade Blue dextran). In UBC (ubiquitin-C)-GFP→wild-type animals, all types of circulating cells other than red blood cells are labeled (**bottom**, green, GFP). Location of insets (on **right**) are indicated with white boxes. **B**, Number of cells within the image volume (230×230×40 µm, centered on microhemorrhage) over time for the same genetically-labeled cell populations shown in (**A**; **left**). The same data were broken down by cell location perivascular (defined as the cell touching the outside of the vessel; **middle**) and parenchymal (**right**) locations (*Cx3cr1*^*GFP/+*^→wild-type: n=12 in 4; *CCR2*^*RFP/+*^: n=6 in 3; UBC→wild-type: n=7 in 2). **C**, In *Cx3cr1*^*GFP/+*^ animals receiving a wild-type bone marrow transplant (wild-type→*Cx3cr1*^*GFP/+*^), labeled cells are nearly all microglia (green, GFP). Axial projections (40 µm thick) of 2-photon image stacks showing the response of microglia for 2 wk after a microhemorrhage (magenta, intravenously injected Texas Red dextran; green, GFP). **D**, Plot of normalized density of microglia over time after a microhemorrhage for regions at different distance from the lesion (wild-type→*Cx3cr1*^*GFP/+*^: n=5 hemorrhages from 3 mice). All scale bars are 100 µm, except for the insets in (**A**), which are 25 µm. Error bars indicate SD.

We first characterized the response of blood-borne monocytes that tend to invade tissue after injury. It has been reported that in animal models of ischemic and hemorrhagic stroke, monocyte-derived tissue macrophages (Mo/MΦ) are among the most numerous blood-borne immune cells infiltrating the brain lesion.^17,18^ Previous work has suggested there are 2 broad classes of these circulating monocytes, inflammatory monocytes, which express CCR2 and exhibit phagocytic, proteolytic, and inflammatory functions, and patrolling monocytes, which express CX3CR1 and tend to attenuate inflammation and participate in wound healing.^19^ In wild-type mice receiving bone marrow from *Cx3cr1*^*GFP/+*^ mice (*Cx3cr1*^*GFP/+*^→wild-type mice), primarily patrolling monocytes were labeled. We observed a small number of GFP (green fluorescent protein)–positive cells (CX3CR1^+^ Mo/MΦ) in the brain tissue near the microhemorrhage beginning a few days to weeks after the lesion (Figure 2A; top), which were nearly all in perivascular locations adjacent to the vessel wall (Figure 2B; right). In mice with inflammatory monocytes labeled with RFP (red fluorescent protein; *Ccr2*^*RFP/+*^), we observed the presence of a few labeled cells (CCR2^+^ Mo/MΦ) near the injury beginning 1 to 2 days after the lesion (Figure 2A; middle), divided about equally between parenchymal and perivascular locations (Figure 2B). Finally, we imaged mice with all leukocytes labeled (UBC [ubiquitin-C]-GFP→wild-type) and again observed the presence a small number of labeled cells in the brain tissue near the microhemorrhage (Figure 2A; bottom), divided about equally between parenchymal and perivascular locations (Figure 2B). Small, punctate, autofluorescent spots also appeared over a few days after the lesion. Taken together, these data suggest that a modest number of blood-borne cells seem after a CMB, with more inflammatory CCR2^+^ Mo/MΦ cells present earlier and more CX3CR1^+^ Mo/MΦ cells present later. Although there were only a few such blood-derived inflammatory cells present after the microhemorrhage, the time course of the appearance of CCR2^+^ and CX3CR1^+^ cells was similar to that seen after larger ischemic lesions, where blood-borne cells play a larger role.^20^

We next determined the role of brain-resident microglia, cells that are known to surveil adjacent tissue by constantly extending and retracting processes,^21^ and to respond to local injuries.^22^ Without an injury, we saw no significant change in microglia location over weeks (imaged in *Cx3cr1*^*GFP/+*^ mice) although the spatial pattern made by their processes changed significantly, consistent with surveillance behavior^21^ (Figure IA in the online-only Data Supplement). However, in the presence of a microhemorrhage, microglia extended processes toward the microhemorrhage within minutes (Figure IB in the online-only Data Supplement). In *Cx3cr1*^*GFP/+*^ mice receiving bone marrow transplant from wild-type mice (wild-type→*Cx3cr1*^*GFP/+*^), microglia were GFP labeled (as well as a reduced fraction of patrolling monocytes because of incomplete chimerism). The density of GFP-labeled cells within 100 µm of bleed increased for the first 2 days (Figure 2C and 2D). There was a trend toward decreased density of labeled cells at distances of 100 to 200 µm away from the injury at 1 day, which recovered by 2 days (Figure 2D).

**Figure 3.**
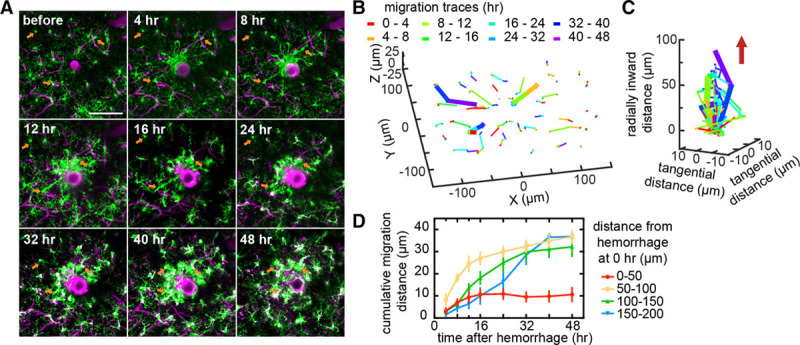
Local increase in microglia density was because of migration of nearby microglia toward the injury. **A**, Axial projections of 40 µm thick 2-photon image stacks over time after a microhemorrhage in a *Cx3cr1*^*GFP/+*^ mouse. **B**, Migration paths of the microglia from (**A**). The color of the segment indicates the time span when that migration occurred. The 3 bold paths correspond to the 3 cells identified with orange arrows in (**A**). **C**, Data from (**B**) plotted with the radial migration toward the lesion from the initial location of each microglia on the *z* axis. The red arrow indicates the direction of the microhemorrhage. The color of the segment in (**B**) and (**C**) indicates the time span when that migration occurred. **D**, Cumulative radial migration distance toward the microhemorrhage for microglia with different initial distances from the target vessel (n=4 hemorrhages from 3 mice). All scale bars are 100 µm. Error bars indicate SD.

**Figure 4.**
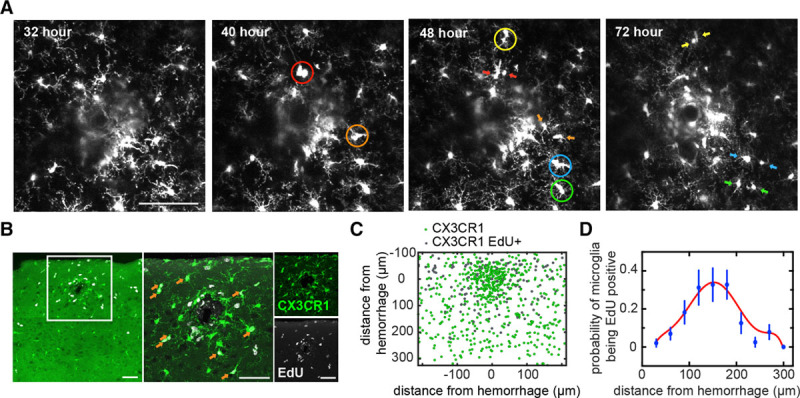
Proliferation of microglia was observed in a shell-shaped region surrounding the lesion. **A**, Axial projections of 40 µm thick 2-photon excited fluorescence image stacks over time after a microhemorrhage in a *Cx3cr1*^*GFP/+*^ mouse. Colored circles indicate cells that will proliferate by the next time point. Arrows with matching colors indicate the pair of daughter cells. Scale bar is 100 µm. The full time series of this particular hemorrhage is shown in Figure IIA in the online-only Data Supplement. **B**, Images of a coronal histological section that intersected a microhemorrhage from an animal euthanized 2 days after the lesion and with repeated 5-ethynyl-2′-deoxyuridine (EdU) injections. Cells that proliferated over those 2 days are labeled with EdU (white) while microglia are labeled with GFP (green fluorescent protein; green). The white box in the **left** image indicates the region for the magnified images to the right. **C**, Composite map of the location of all microglia and EdU+ microglia from coronal sections across 17 hemorrhages from 3 mice, with the location of the microhemorrhage centers aligned at (0, 0). The cortical surface is at the **top** of the image. **D**, The probability of microglia being EdU positive as a function of radial distance from the microhemorrhage from the data shown in (**C**) and a polynomial fit (red trend line). All scale bars are 100 µm.

We next evaluated the relative role of migration and proliferation in the microglia density increase near the microhemorrhage. We followed the movement of individual microglia for 48 hours after the lesion in *Cx3cr1*^*GFP/+*^ mice (Figure 3A and 3B). Microglia migrated radially inward toward the lesion, with some cells traveling distances of up to 40 µm (Figure 3C). Microglia initially within 100 µm of the target vessel began to migrate within hours while cells starting further from the lesion took progressively longer to begin migrating (Figure 3D). Migration speeds ranged from 0.5 to 2 µm/h. In microglia that migrated, we observed a stereotypical temporal sequence where the cell first extended processes toward the lesion, and then these processes became thicker, and finally the cell body migrated along the path of the thickened process toward the lesion, catching up to the process that was previously extended (Figure IB in the online-only Data Supplement). We also observed proliferation of some microglia using time-lapse 2-photon excited fluorescence imaging at times 40 hours or later after the injury, as evidenced by the presence of 2 microglia at the location of a single, larger microglia from the previous time point (Figure 4A; Figure II in the online-only Data Supplement). To quantify the role of proliferation in the microglia density increase, we used 5-ethynyl-2′-deoxyuridine labeling and immunohistology approaches (Figure 4B; Figure III in the online-only Data Supplement). Interestingly, we found microglia proliferated predominantly in an ≈100 µm wide shell with an ≈175 µm radius that surrounded the targeted penetrating arteriole (Figure 4C and 4D). Taken together, these data suggest that microglia near a microhemorrhage respond by rapidly migrating toward the injury, leading to a slight decrease in density in the surrounding neighborhood. After ≈40 hours, microglia then proliferate in this region where the density had decreased. This pattern of migration and proliferation leads to increased density near the lesion while preserving microglia density farther away. After 2 days, the microglia density close to the injury began to slowly decline and neared baseline density by 2 weeks, with microglia regaining a more ramified morphology.

**Figure 5.**
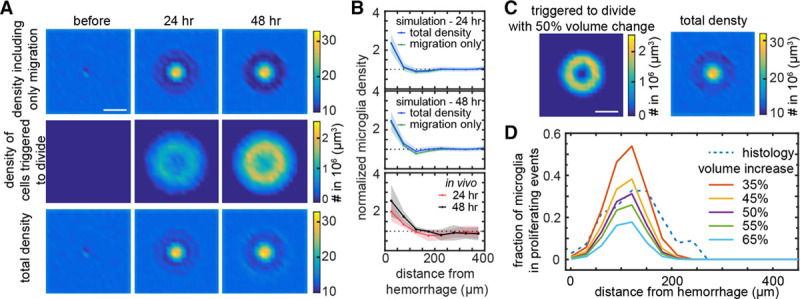
Simulation of microglia density changes because of migration and proliferation after a microhemorrhage. **A**, Map of the density of microglia including only migration and not proliferation in the simulation (**top**), of microglia committed to division (**middle**), and of total microglia density (**bottom**) at baseline and at 24 and 48 hours after the microhemorrhage. **B**, Plot of normalized density of microglia as a function of distance from a microhemorrhage at 24 (**top**) and 48 (**middle**) hours after the injury for the simulation showing the density including migration and proliferation (blue) or just migration (green). The lower plot shows experimental in vivo measurements at 24 (red) and 48 (black) hours after the lesion (same data as shown in Figure 3B; n=5 hemorrhages from 3 mice). Lines (shading) indicate mean (SD). **C**, Map of the total density of microglia (**left**) and of the density of microglia committed to division (**right**) at 48 hours after a microhemorrhage using models where microglia proliferation occurs when the domain volume of a microglia increases by >50% percentage. **D**, The fraction of microglia that have either committed to divide or are daughter cells of a proliferation event as a function of distance from and at 48 hours after the microhemorrhage for different density-dependent microglia proliferation models. The dashed line represents the fraction of microglia that were found to be 5-ethynyl-2’-deoxyuridine positive in experiments (same data as in Figure 4D). Scale bars are 200 µm.

The spatial overlap between the regions of microglia density decrease and of microglia proliferation suggests that microglia may be triggered to proliferate when their local density decreases. To explore this idea, we computationally simulated the response of microglia to a microhemorrhage based on the migration rates of microglia measured by in vivo imaging (data from Figure 3; *Cx3cr1*^*GFP/+*^) and the proliferation probabilities that were assessed histologically (data from Figure 4D; *Cx3cr1*^*GFP/+*^) to determine whether we could account for the microglia density changes near the lesion. First, we used our experimental measurements to extract probability distributions for microglia migration distances (Figures IV and V in the online-only Data Supplement) and microglia proliferation (Figure 4D), each as a function of distance from and time after the microhemorrhage. The resulting simulations showed an increase in microglia density near the lesion for 48 hours that agreed well with the spatially-dependent microglia density measured in separate experiments (data from Figure 2C and 2D, wild-type→*Cx3cr1*^*GFP/+*^), validating this simulation approach (Figure 5B). A surrounding shell-shaped region with a slightly decreased microglia density at 24 hours was also evident in the simulations and filled in because of proliferation by 48 hours (Figure 5A and 5B). To evaluate whether the spatial pattern of microglia proliferation could be recreated based on changes in the local microglia density, we next ran the simulation using experimentally-measured microglia migration distributions but now assuming that microglia were triggered to proliferate if their domain volume increased by more than a defined factor (Figure 5C; Figure VI in the online-only Data Supplement). We found that the simulation predicted the observed spatial distribution of microglia proliferation using a 50% volume increase as the threshold value to trigger a proliferative event (Figure 5D).

**Figure 6.**
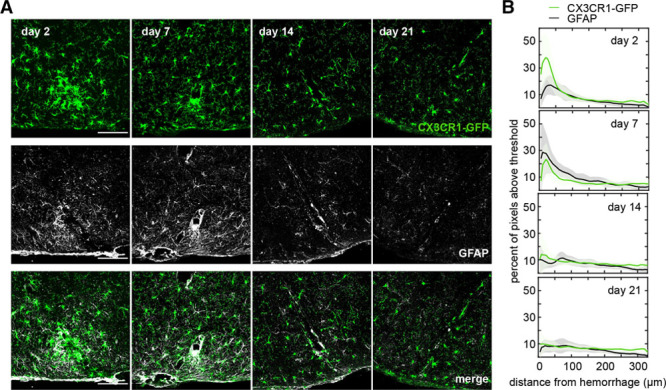
Astrocyte activation was observed in the region of microglia density increase but peaked at later times. **A**, Images of coronal histological section that intersected a microhemorrhage from animals euthanized 2, 7, 12, and 21 days after the lesion. Microglia were visualized by the expression of CX3CR1-GFP (green fluorescent protein; **top**), astrocytes were visualized by immunolabeling of GFAP (glial fibrillary acidic protein; **middle**). The **bottom** shows an overlay. Cortical surface is to the **bottom** of the images. **B**, The percent of image pixels above a threshold as a function of distance away from the lesion and over time for microglia and astrocytes. Data represent the average of 6 to 9 hemorrhages across 2 to 3 mice for each time point. Scale bars are 100 µm.

We also sought to understand the participation of astrocytes in the inflammatory response after a CMB. Although the representation of astrocyte reactivity by the expression level of GFAP (glial fibrillary acidic protein) is still debatable, this approach is widely used. After creating laser-induced microhemorrhages in *Cx3cr1*^*GFP/+*^ mice, we euthanized mice at different time points, immuno-stained for GFAP, and imaged GFAP expression, as well as GFP-labeled microglia. We saw extensive expression of GFAP around the lesion starting at 2 days and peaking at 7 days after the injury (Figure 6A). Over time, GFAP expression declined, and we did not observe the formation of an astrocytic glial scar near the lesion at later time points (Figure 6B). Consistent with our in vivo imaging, GFP labeling density was highest at 2 days after the injury and declined afterward, indicating that the peak response of astrocytes was delayed relative to that of microglia.

## Discussion

The presence of CMBs is highly correlated with cognitive impairment and functional decline.^1,2,8,9^ However, in previous studies with the laser-induced microhemorrhage model used here, we found that these small bleeds cause neither the death of nearby neurons nor the degeneration of their dendritic arbors.^11^ We also found that although neurons near the injury initially lost their ability to respond to a stimulus, they recovered within hours.^12^ We did find that microhemorrhages led to a rapidly-initiated inflammatory response, as indicated by an increased density of CX3CR1^+^ cells in the vicinity of the lesion, that was sustained for weeks.^11^ In this work, we used a series of in vivo imaging and histological studies to follow the behavior of several different inflammatory cell types after the induction of ≈100 µm sized hemorrhages in healthy adult mice. The initial inflammatory response was dominated by brain-resident microglia, which exhibited a coordinated pattern of migration and proliferation that led to an increased density of microglia near the lesion over hours to days (primarily by migration from the surrounding tissue) while later recovering the density of microglia in the surrounding tissue (by proliferation). We also found reactive astrocytes over a similar spatial region as microglia but with the response delayed by a few days relative to microglia. The delayed astrocyte activation could be explained by the recent finding that activated, inflammatory microglia induce astrocyte activation by releasing cytokines.^23^ A small number of Mo/MΦ were also identified in the brain near the lesion, with CCR2^+^ Mo/MΦ found in the first couple of days, and CX3CR1^+^ Mo/MΦ found later, over days to weeks after the injury.

There are several mechanisms by which the inflammation we observe after a microhemorrhage could impact neural function. In healthy brain, microglia and astrocytes play an active role in remodeling synaptic circuits.^24,25^ However, in response to inflammatory stimuli, this healthy glial-mediated rewiring can be altered. For example, under ischemic stress microglia exhibited prolonged contact with synapses and were found to phagocytize synaptic components.^25^ Moreover, activation of microglia and astrocytes results in the secretion of cytotoxic factors, cytokines, chemokines, growth factors, and even neurotransmitters that tune inflammatory cell function, recruit additional leukocytes, and may also be neurotoxic and interfere with neuronal function.^26–29^ Infiltrated monocytes may also secrete factors that modulate learning and memory.^29^ Thus, activation and redistribution of inflammatory cells near a CMB, the phagocytic activity of these cells, and the signaling factors they secrete all likely affect the behavior of nearby neurons and other cells. The accumulation of such events may contribute to the cognitive effects associated with CMBs.

In the healthy brain, microglia are intercalated throughout the entire parenchyma and are arranged in nonoverlapping domains, which they surveil by extending and retracting processes.^21^ Through this surveillance, microglia detect subtle changes in the microenvironment and react to a broad variety of insults, sensed with an array of surface receptors, including purigenic^22^ and fractalkine^30^ receptors. Like other tissue-resident macrophages, microglia maintain their population locally by proliferative self-renewal,^31–34^ with sporadic proliferation under normal conditions and clonal expansion under prolonged and severe pathological conditions.^34^ Here, through a series of in vivo analyses, we describe the concerted spatiotemporal response of microglial migration and proliferation in a focal injury model. We observed proliferation of microglia predominantly in regions where the density of microglia declined because of migration of other microglia toward the lesion, suggesting that microglia may sense their local density and proliferate when the density decreases. It is not clear how microglia might sense such changes in density, but it has been reported that microglial processes repel one another when they make contact.^21^ Proliferation occurred primarily at a region and time relative to the CMB in which microglia had a ramified morphology, suggesting that it is the resting-state microglia, rather than the activated microglia responding to an injury, that tend to proliferate. This proliferation compensated for the decreased microglia density because of migration toward the injury. This coordinated pattern of migration and proliferation suggests a way that microglia may maintain their characteristic organization and cellular density in nonoverlapping domains after a nearby focal injury. Further work needs to be done to uncover the specific molecular mechanisms underlying this coordinated pattern of migration and proliferation in microglia, as well as to follow the fate of activated microglia after the injury.

Our study elucidated the response of a subset of inflammatory cells after a femtosecond laser-induced microbleed, but our work leaves some aspects of the inflammatory response unresolved. We characterized the time course of the appearance of CX3CR1^+^ and CCR2^+^ Mo/MΦ after a microhemorrhage, but we studied the response of these cells in separate reporter mice, preventing us from elucidating the role of phenotype switching in Mo/MΦ cells. In a focal ischemic model, it has been reported that many CCR2^+^ Mo/MΦ that had invaded after the stroke later transdifferentiated to CX3CR1^+^ Mo/MΦ^20^. Our data align with these findings in that CCR2^+^ Mo/MΦ are the majority blood-derived cell type present early after injury, and CX3CR1^+^ Mo/MΦ are more dominant at later time points. It remains unclear, however, whether CCR2^+^ Mo/MΦ switched to CX3CR1^+^ Mo/MΦ or whether CX3CR1^+^ Mo/MΦ separately invaded. In addition, we cannot rule out the invasion of other blood-borne leukocytes although their numbers would likely be low given that the total number of labeled cells found in UBC-GFP→wild-type bone marrow transplant mice approximately matched the sum of cells found in CX3CR1^GFP/+^→wild-type bone marrow transplant mice and CCR2^RFP/+^ mice. In addition, endogenous brain cells other than microglia and astrocytes, such as oligodendrocytes and perivascular macrophages, could have also participated in the initiation and resolution of inflammatory responses caused by laser-induced microhemorrhages, all of which needs further work. Finally, our work was conducted in healthy adult animals, and the cascade of inflammatory response may differ considerably in older mice^35^ or in animals with comorbidities, such as cerebral amyloid angiopathy.^36^

## Conclusions

Here in this work, a single event of bleeding from a penetrating arteriole induced an inflammatory response that spanned more than a week and was characterized by microglia and later astrocyte activation, as well as the presence of monocyte-derived tissue macrophages of different phenotypes. Although this small bleed does not cause direct neuronal degeneration^11^ nor loss of responsiveness to external stimuli,^12^ this prolonged inflammatory response could cause subtle modulation of normal brain function, and the accumulation of such events could underlie cognitive decline.

## Acknowledgments

Confocal images were acquired through the Cornell University Biotechnology Resource Center, with New York State Stem Cell Science (CO29155) and National Institutes of Health (S10OD018516) funding for the shared Zeiss LSM880 confocal/multiphoton microscope. We thank Sylvie Allen and Kevin Yager in the Cornell Center for Animal Resources and Education for assistance with the mouse irradiation for bone marrow transplants.

## Sources of Funding

This work was supported by the National Institutes of Health grants NS080098 (Dr Schaffer), NS081179 (Dr Anrather), and American Heart Association grant 16PRE27600010 (Dr Ahn).

## Disclosures

None.

## Supplementary Material
