# Supplementary material for: Diverse Inflammatory Response After Cerebral Microbleeds Includes Coordinated Microglial Migration and Proliferation
Source: Stroke. 2018 May 29;49(7):1719–26. doi: 10.1161/STROKEAHA.117.020461 (PMC6019563; doi:10.1161/STROKEAHA.117.020461)
Supplement: Supplementary file 1 [file str-49-1719-s001.pdf]

ONLINE SUPPLEMENTAL MATERIAL

The diverse inflammatory response after cerebral microbleeds includes coordinated microglial migration and proliferation

[1]Sung Ji Ahn, MS; [2]Josef Anrather, DVM; [1]Nozomi Nishimura, PhD; *[1]Chris B. Schaffer, PhD

[1]Meinig School of Biomedical Engineering, Cornell University, Ithaca NY 14853

[2]Feil Family Brain and Mind Institute, Weill Cornell Medical College, New York NY 10065

Supplemental Methods

Animals Experiments were performed in 20 to 40 week old transgenic mice on a C57BL/6J genetic background. Breeders were purchased from The Jackson Laboratory (CX3CR1-GFP #8451[1], UBC-GFP #4353[2], CCR2-RFP #17586[3], Thy1-YFPH #3782[4], wild-type) and animals were crossed and/or received bone marrow transplants to fluorescently label targeted cell types. All animal experiments were conducted in strict accordance with the recommendations in the Guide for the Care and Use of Laboratory Animals published by National Institutes of Health, and all animal procedures were approved by the Cornell University Institutional Animal Care and Use Committee (protocol numbers 2009-0043 and 2015-0029).

Bone marrow transplantation and chimerism Bone marrow chimeras were generated from transgenic and wild-type mice. Six to eight week old recipient mice were anesthesized with ketamine and xylazine (10 mg/100 g mouse weight ketamine; 1 mg/100 g xylazine; intraperitoneally). Mice were placed in a custom-built lead head shield and exposed to a lethal dose of gamma irradiation (10 Gy). Donor mice (2-4 months old) were deeply anesthetized using isoflurane (~3% in oxygen). Both femurs were removed and the animals euthanized by cervical dislocation. Bone marrow cells were flushed out from the femur with sterile saline and the density of cells was measured using a hemocytometer. Recipient mice received ~10^7 donor bone marrow cells through retro orbital injection 1-3 hr after irradiation. Nine weeks after the bone marrow transplant, ~100 μL of blood was collected from submandibular bleeding and the degree of chimerism was assessed by flow cytometry. We only used mice in which the percentage of circulating white blood cells that came from the donated bone marrow was more than 63%. Although the head shielding we used here reduces the degree of chimerism achieved with the bone marrow transplant, it avoids gamma irradiation of the head, which could drive brain inflammation that may confound interpretation of the results[5, 6].

Chronic cranial window preparation Optical access to brain was achieved through a long-term implanted glass-covered cranial window, as described previously[7]. Animals were anesthetized using isoflurane (1.5–2% in oxygen) and placed on a feedback controlled heating blanket that maintained body temperature at 37˚C (50-7053P; Harvard Apparatus). Mice were given either glycopyrrolate (0.05 mg/100 g mouse weight; intramuscularly; Baxter, Inc.) or atropine sulfate (0.005 mg/100 g; subcutaneously; 54925-063-10, Med-Pharmex Inc.) to prevent lung secretions. Animals were also given dexamethasone (0.025 mg/100 g; subcutaneously; 07-808-8194, Phoenix Pharm Inc.) and ketoprofen (0.5 mg/100 g; subcutaneously; Zoetis Inc.) to reduce post-surgical inflammation and pain. A 6-mm diameter bilateral craniotomy was performed over parietal cortex using a dental drill. The exposed brain was covered with sterile saline and sealed with an 8-mm diameter glass coverslip using cyanoacrylate glue (Loctite 495; Henkel), tissue adhesive (70200742529, 3M™), and dental cement (Co-Oral-Ite Dental Mfg Co.). Animals were given a single injection of 5% weight/volume (w/v) glucose in saline at the conclusion of surgery (1 ml/100 g). All mice recovered at least 21 days from this surgery before in vivo imaging and microhemorrhage induction. A small number of animals had poor clarity cranial windows and were excluded from further study. There were no other exclusion criteria in any experiments in this study.

Multi channel *in vivo* two-photon excited fluorescence microscopy Mice were anesthetized with isofluorane (1.5–2% in oxygen) and placed in a stereotaxic apparatus equipped with a feedback-controlled heating pad. As above, mice were given either glycopyrrolate (0.05 mg/100 g;

intramuscularly) or atropine sulfate (0.005 mg/100 g; subcutaneously) to prevent lung secretions. The blood plasma was labeled with either red or blue fluorescent dextran-conjugated dyes (2.5% w/v Texas-Red 70 kDa used with GFP positive cells, D-1830; 5% w/v Cascade-Blue 10 kDa used with RFP positive cells, D-1976; Invitrogen) diluted in sterile saline and retro-orbitally injected (50 μl) before imaging. Imaging was performed on a custom-built four-channel 2PEF microscope using the following emission filters (center wavelength/bandwidth): 417/60 (Cascade-Blue), 494/41 (GFP), 550/49 (YFP) and 641/75 nm (RFP and Texas-Red) separated with long-pass dichroics with cutoffs at 458, 520 and 562 nm. Excitation pulses came from a tunable Ti:Sapphire laser (Vision II, Coherent) set to a wavelength of 880 nm for imaging YFP, GFP, Texas-Red and to 800 nm for Cascade-Blue. An additional femtosecond laser at 1030 nm was used to image RFP (Yg fiber oscillator/amplifier; Satsuma; Amplitude Système). Image stacks were acquired through ScanImage software (version 3.8, Vidrio Technologies)[8]. Respiration was monitored throughout the imaging session and the isoflurane level was adjusted to maintain a steady breathing rate of ~1 Hz. During the imaging session, mice received an hourly dose of 5% w/v glucose in saline (1 ml/100 g). In animals that received atropine and not glycopyrrolate, the atropine was supplemented hourly (0.001 mg/100 g; subcutaneously). For time-lapse imaging, animals were woken up between each subsequent imaging session.

Induction of microhemorrhage by femtosecond laser ablation Microhemorrhages were produced in the descending segment of penetrating arterioles (PAs) as previously described[9-11]. Femtosecond laser pulses were tightly focused (20x, 1.0 numerical aperture (NA), water immersion, cover glass corrected objective; W Plan-Apochromat (Zeiss)) on the outer edge of the vessel lumen of the targeted PA. We used 50-fs duration laser pulses produced by a Ti:sapphire regenerative amplifier (Legend 1 k USP; Coherent), pumped by a Q-switched laser (Evolution 15; Coherent) and seeded by a Ti:Sapphire oscillator (Chinhook; Kapteyn-Murnane Laboratories Inc.) that was pumped by a continuous wave laser (Verdi-V6; Coherent, Inc). Laser energy was controlled using neutral density filters. We began with a laser energy of ~50 nJ and targeted the PA about 100 μm beneath the cortical surface. A burst of 100 laser pulses (1 kHz repetition rate) was applied while we watched for extravasation of fluorescently-labeled blood plasma from the targeted vessel. If the vessel did not rupture then a burst of 200 and then 300 pulses with the same energy was applied. If the vessel still did not rupture, the laser pulse energy was increased by 50% and this process was repeated. This procedure ensured that the minimum laser energy required to rupture the targeted vessel was used to induce a microhemorrhage. Typical laser energies used to rupture PAs varied from 95 to 240 nJ, largely dependent on the depth at which the vessel was targeted and the clarity of the cranial window. We acquired 2PEF image stacks with 1 μm axial spacing before and after inducing the hemorrhage, as well as movies of the hemorrhage induction process (e.g. Supplementary Movie).

Our preliminary in vivo imaging revealed that each microhemorrhage caused changes in microglia density only within about 300 μm of the lesion. Therefore, we typically placed one to two microhemorrhages in each hemisphere, separated by at least 600 μm, and also imaged one control region at least 1 mm away from any microbleed. For the studies in CCR2+ mice, in which a small number of animals were available, a second round of hemorrhages was made about two months after the first set, also at distances of at least 600 μm from the previous lesions. In all other animals, only a single set of microhemorrhages was induced. For the histological studies of microglia proliferation (Fig. 4) and of astrocyte reactivity (Fig. 6), we placed more hemorrhages in each hemisphere, while maintaining a minimum distance of at least 600 μm between lesions. For the histological studies, we only used hemorrhages we could unambiguously link from the in vivo images to the specific histology slice, and

increasing the number of hemorrhages increased the chance of reliably identifying tissue slices of interest.

Analysis of cell dynamics after a microhemorrhage For each set of experiments, transgenic or chimeric mice expressing fluorescent protein were used. In each mouse, we made one or two microhemorrhages and imaged one control region. We manually identified the location and numbers of cells of interest in a 40-µm thick slab, centered at the depth of the microhemorrhage, across these imaging times and locations. In case of determining the location of infiltrating cells, we further classed them as being perivascular if they surrounded or made contact with the target vessel and as being parenchymal if they did not. To assess migration of microglia between different time points, we used the Rigid Registration plugin in FIJI to align all image stacks, using blood vessels and/or dendritic arbors (in animals with *Thy1-YFP*) as alignment guides. Because not all microglia migrated and because those that did tended to not migrate very far (<25 µm) between time points, it was possible to identify each individual microglia across each imaging time point to determine their migration paths. We also observed microglia proliferation, characterized by an elongated cell body shape and then two daughter cells at that location in the next imaging session (and with all neighboring microglia accounted for).

Post-mortem Immunohistochemistry Animals were transcardially perfused with 15 ml of phosphate buffered saline (PBS) (Sigma-Aldrich) followed by 30 ml of 4% w/v paraformaldehyde (PFA) (Fischer Scientific) in PBS. Brains were extracted, stored overnight in in 4% PFA in PBS at 4°C, then in 15% w/v sucrose in PBS for 8 hr at 4°C, followed by 24 hr in 30% sucrose, and then in 60% sucrose until sectioning. The microhemorrhage locations were known relative to the large surface vessels from 2PEF images and those large vessels were readily visualized on the post-mortem brain surface after brief Toluidine Blue staining. The tissue was then frozen and cut into 20-µm thick coronal sections on a cryostat, mounted onto microscope slides, and the sections that went through a microhemorrhage identified. To assess microglia proliferation, we placed microhemorrhages in mice as described above and then gave intraperitoneal injections of 5-ethynyl-2'-deoxyuridine (EdU; 2 mg/100 g) every 8 hr for two days. After the 6th injection, mice were sacrificed for histological analysis. We used a kit to detect EdU-labeled cells (Click-iT EdU, C10340, Invitrogen) in the tissue sections. For immunohistochemistry to detect Ki67 and GFAP expression, the following antibodies were used: rabbit anti-Ki67 monoclonal antibody at 1:100 dilution, (GTX16667, GeneTex) with a goat anti-rabbit Alexa flour 594 conjugated secondary antibody at 1:200 dilution (A-11037, Invitrogen) and chicken anti-GFAP polyclonal antibody at 1:1000 dilution (ab4674, abcam) with a goat anti-chicken Alexa flour 594 conjugated secondary antibody at 1:200 dilution (A-11042, Invitrogen). Stained slides were mounted using an anti-fade mounting medium that contained DAPI (Vetashield, H-1200, Vector Laboratories). Fluorescent images were collected on an inverted confocal fluorescence microscope (LSM880, Zeiss) using a 40x/1.1 NA long working distance water immersion objective (421767-9971-711, Zeiss).

Modeling microglia density change after microhemorrhage We developed a simple model of the microglia response to a brain microhemorrhage using our data on microglia migration and proliferation to account for the density change we observed in microglia over two days after the injury. *In vivo* measurements from 11 *Cx3cr1*$^{GFP/+}$ mice revealed a microglia density of $1.3 \pm 0.3 \times 10^4$ cells/mm^3, and a minimum nearest neighbor distance between microglia of 37±11 µm. In our simulation, we first seeded microglia at random locations in an 800 × 800 × 50 µm volume, while matching the cell density and spacing measured from image stacks taken before inducing microhemorrhages. We used *in vivo* measurements of microglia migration after the microhemorrhage (Fig. 3) to calculate a probability

distribution of the radial migration distance for microglia at different distances from and times after the microhemorrhage (Supplementary fig. 4; smoothed over 20 µm in distance from the lesions and 5 µm in migration distance). This probability distribution was used to determine the radial migration of each microglia every 4 hr for 16 hrs and then every 8 hr until 48 hr after the lesion. From our *in vivo* imaging, we also quantified the minimum nearest-neighbor microglia spacing as a function of distance from and time after the lesion (Supplementary fig. 5; smoothed over 20 µm in distance from the lesions and 5 µm in migration distance). If the migration of a microglia placed it closer to another microglia than one standard deviation below the mean of this nearest-neighbor microglia spacing then we determined a new migration distance for that cell.

The proliferation probability and the time for microglia cell division was determined from the number of EdU and Ki67 positive microglia in the histology images (Fig. 4). Only cells that were actively in the cell cycle when the animal was sacrificed would express Ki67. With the EdU dosing strategy we used, all cells that had entered the cell cycle over the full 48 hours after the injury, as well as all daughter cells, would be labeled. The number of Ki67, N_{Ki67}, and EdU positive cells, N_{EdU}, at a time, T, after the microhemorrhage are thus given by:

$$N_{Ki67}(r) = \sum_{t=\mathrm{T}-\tau}^{\mathrm{T}} R(r) * N * (1 - R(r))^t \qquad \text{Eq. 1}$$

$$N_{EdU}(r) = \sum_{t=1}^{\mathrm{T}-\tau} 2 * R(r) * N * (1 - R(r))^t + \sum_{t=\mathrm{T}-\tau}^{\mathrm{T}} R(r) * N * (1 - R(r))^t \qquad \text{Eq. 2}$$

where N is the number of microglia at baseline, τ is the time required for microglia cell division, $R(r)$ is the probability of a microglia being triggered to proliferate and is dependent on r, the distance from the microhemorrhage, and t is time after the lesion given in discrete steps (hrs). This model assumes that within this time frame (maximum simulation times of T=48 hrs), the daughter cells of a microglia proliferation event do not divide again. We then calculated $R(r)$ and τ to match the spatially dependent fraction of microglia that were EdU positive (Fig. 4D) and the ratio of Ki67 to EdU positive microglia ($N_{Ki67}/N_{EdU} = 0.084$). At each time point in the simulation, we used the distance-dependent probability of a microglia entering the cell cycle, $R(r)$, to identify cells that will later divide. We then seeded a new microglia at a location ~30 µm away after a time, τ. We averaged the results of 1,000 simulations and compared the resulting density profiles to experimental measurements of the density of microglia after a microhemorrhage that were conducted in separate animals (data from Fig 2C and D, in wild-type → Cx3cr1$^{GFP/+}$).

Modeling a causal relationship between microglia density change and proliferation To examine the hypothesis that microglial proliferation was initiated by a density changes, we then used an alternate model for microglia proliferation that depended only on local changes in microglia density. After seeding microglia, we assigned each image voxel to the closest microglia, thereby defining a domain volume for each microglia. We then ran the simulation with microglia migration handled as described above. At each time step, we recalculated the domain volume for each microglia. If the domain volume increased by more than a specified fraction then that cell was flagged to divide and after a time, τ, a new microglia was seeded ~30 µm away.

Image, data, and statistical analysis All 2PEF images were analyzed using FIJI[12]. For the generation of images of fluorescent species with overlapping spectral profiles, we used a custom linear unmixing code written in Matlab. Image manipulations were limited to linear adjustments of image contrast. All displayed images represent maximal projections of 2PEF image stacks except for the single plane movie frames in Fig. 1A and the renderings in Fig. 1B and Supplementary fig. 2B, which used Volocity (PerkinElmer Inc) and Imaris (Bitplane), respectively. For the quantification of fractional volume occupied by microglia and astrocytes as a function of time and distance from the microhemorrhage shown in Fig. 6, we first axially projected the ~10-μm thick confocal image stack, then binarized the image with the highest threshold that preserved the visibility of the fine processes of microglia and astrocytes. The percentage of pixels above threshold was then determined as a function of the radial distance from the microhemorrhage center. Data in all plots represents the mean and error bars represent the standard deviation.

Movie Caption

Supplementary movie 1: Induction of microhemorrhage by targeted rupture of a penetrating arteriole using tightly focused femtosecond laser pulses. Images were taken at ~1 frame per second and are displayed sped up by a factor of 5. The field of view of the movie is 260 μm.

Supplemental Figures and Legends

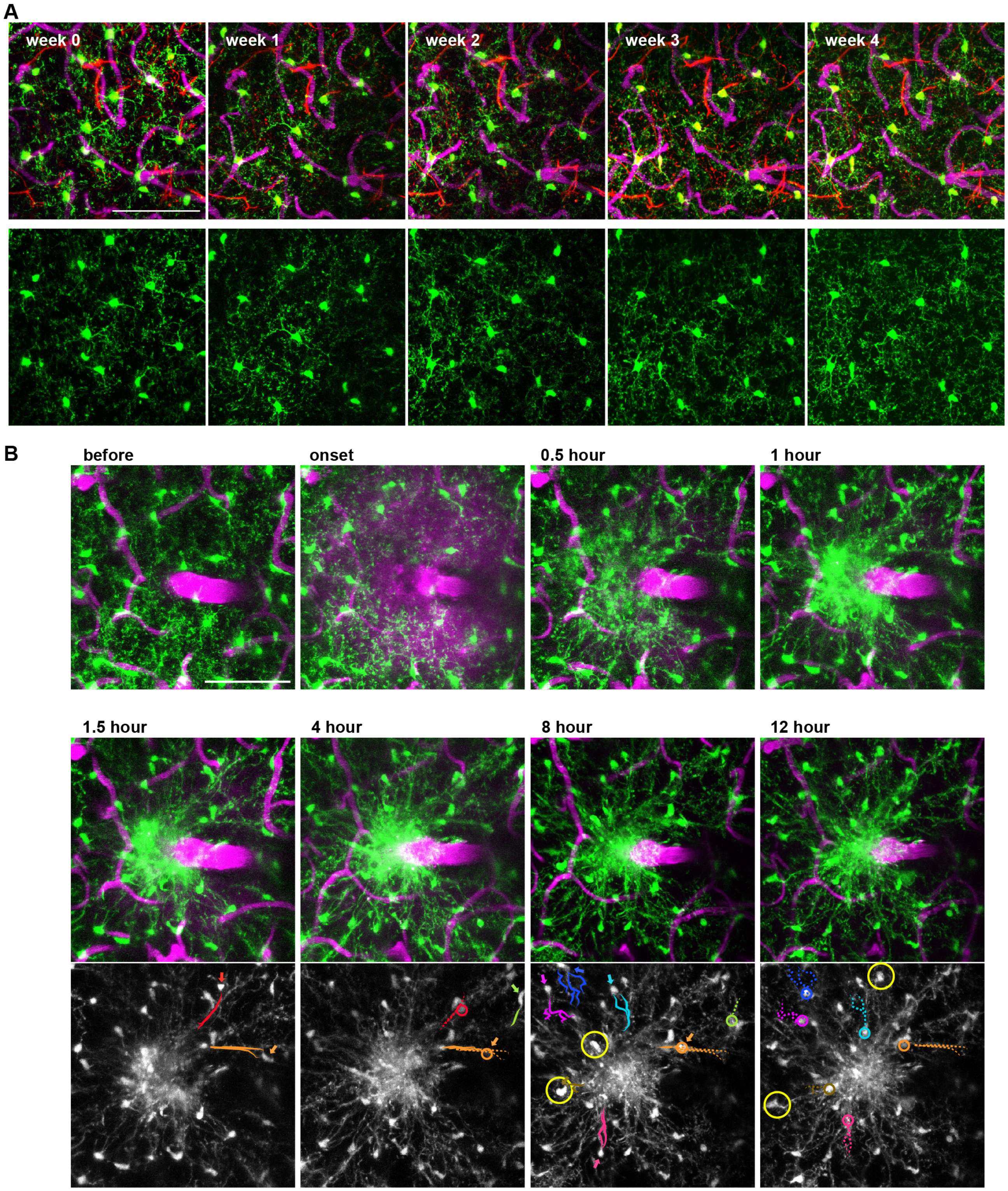

Supplementary figure I: Microglia locations were highly stable in the absence of an injury but immediately responded to a microhemorrhage and migrateed toward the injury following a path

defined by previous processes extension. (A) Axial projections of 50-μm thick 2PEF image stacks over time in a *Cx3cr1*$^{GFP/+}$ mouse taken ~100 μm beneath the cortical surface on the contralateral side of the brain from the location of any microhemorrhage (magenta, i.v. injected Texas Red dextran; green, GFP; red, YFP expressed in Layer V pyramidal neurons). Although the microglial processes differed in spatial arrangement between time points, nearly all microglia in the imaging field retained their approximate position. Scale bar is 50 μm. (**B**) Axial projections of 40-μm thick 2PEF image stacks over time after a microhemorrhage in a *Cx3cr1*$^{GFP/+}$ mouse (magenta, i.v. injected Texas Red dextran; green, GFP). The grey-scale images show just the GFP-labeled microglia for the last four time points. For several cells, the solid colored traces identify major branches of processes that have extended toward the microhemorrhage. At subsequent time points, these cells (position over time indicated by circles with matching color) migrated along the path defined by the extended process. The path the cell migrated along is indicated with dashed lines of matching color. The yellow circles indicate nearby microglia that migrated into the region of interest, either from above or below this region or from the sides. Scale bar is 100 μm.

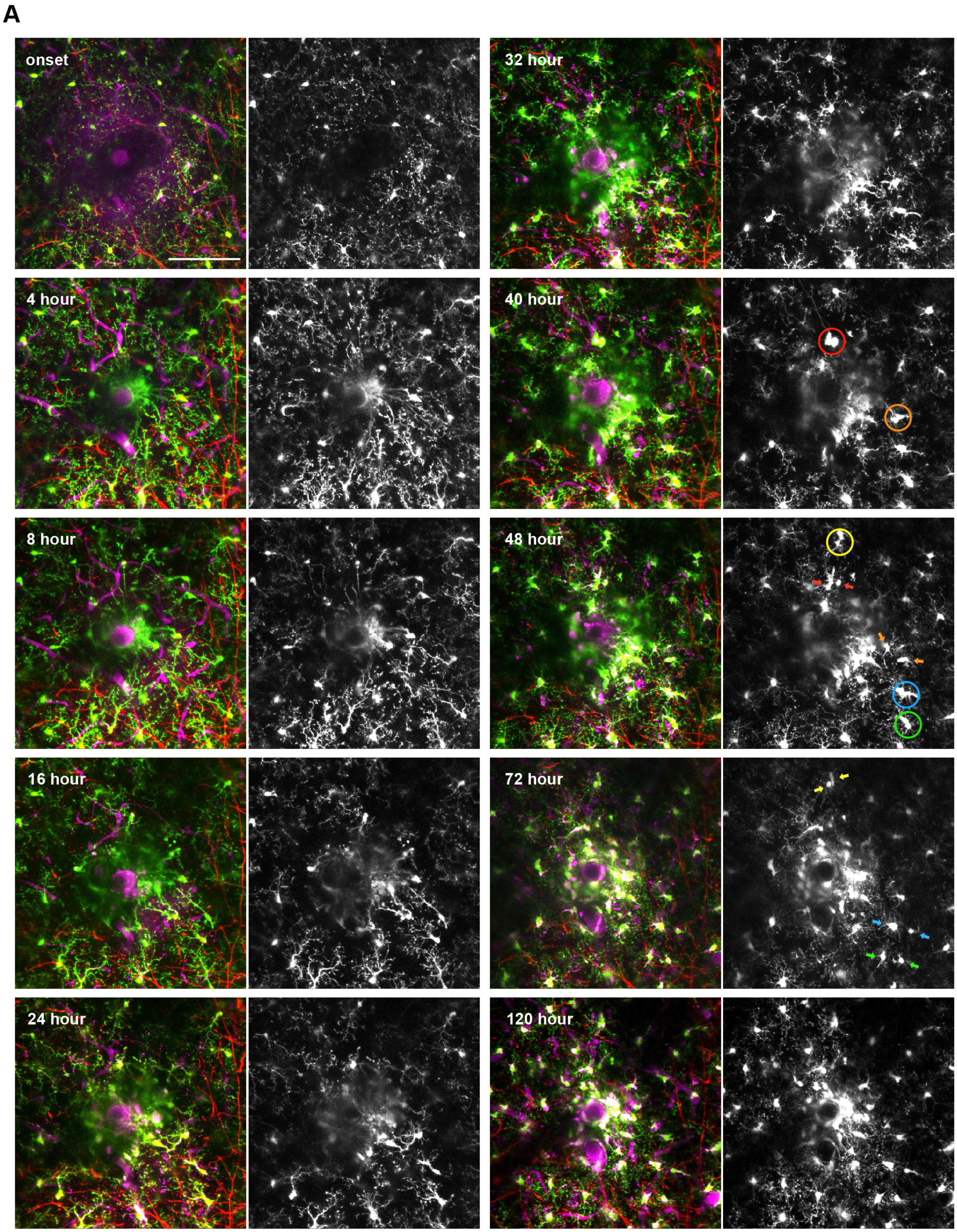
A
onset
4 hour
8 hour
16 hour
24 hour
32 hour
40 hour
48 hour
72 hour
120 hour

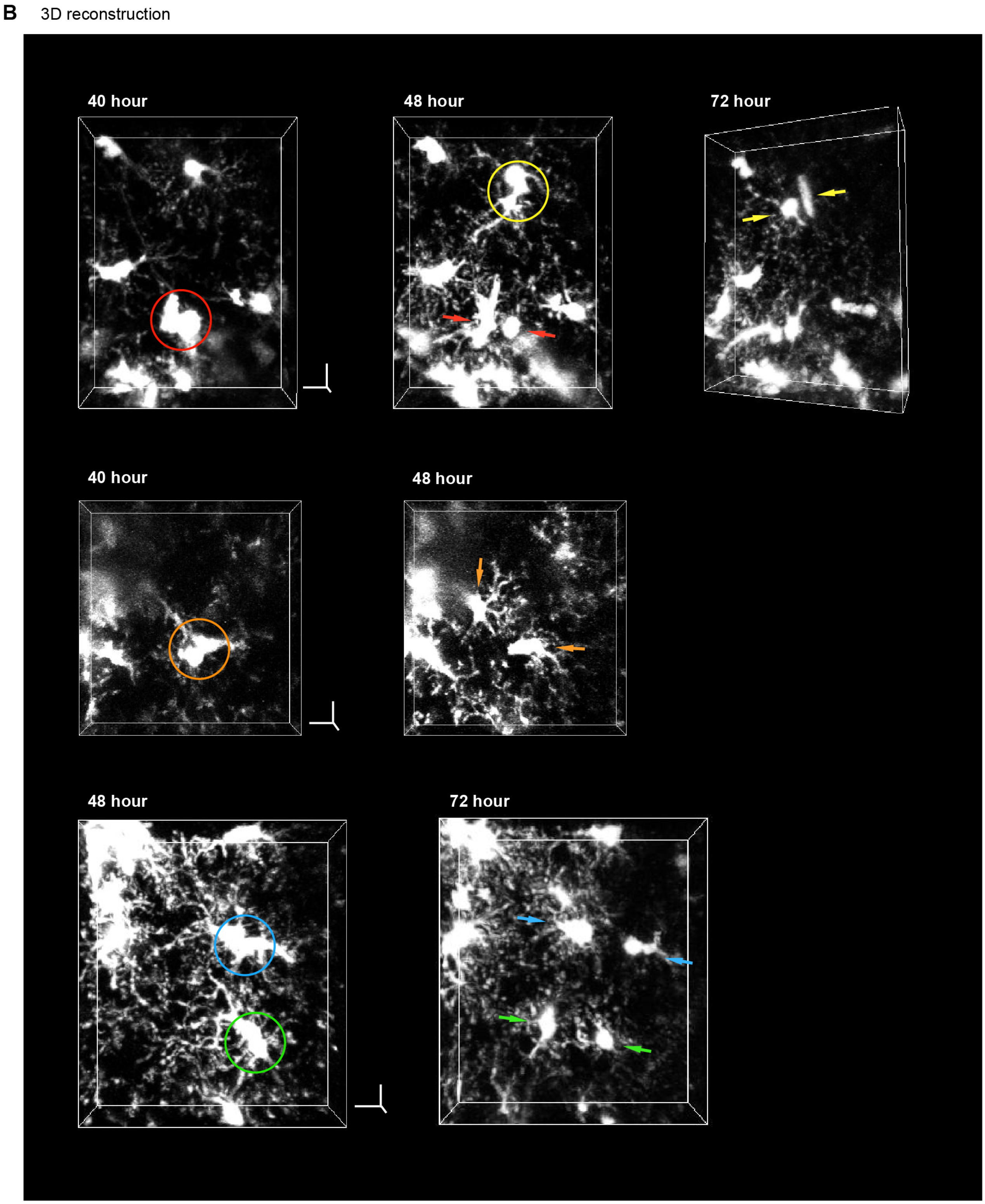

Supplementary figure II: Daughter cells from a microglia proliferation event were observed only more than 40 hours after a microhemorrhage. (**A**) Axial projections of 40-µm thick 2PEF image stacks over time after a microhemorrhage in a *Cx3cr1*$^{GFP/+}$ mouse (magenta, i.v. injected Texas Red dextran; green, GFP; red, YFP expressed under control of the Thy1 promoter to label Layer V pyramidal

neurons and their axons and dendritic arbors, which were visible at this depth). Greyscale images show just GFP-labeled microglia. Colored circles indicate cells that will proliferate by the next time point. Arrows with matching colors indicate the pair of daughter cells. Scale bar is 100 μm. (**B**) Three-dimensional reconstructions of some of the microglia cell division events shown in (A). Colored circles and arrows indicate corresponding cells from (A). Scale bars are 10 μm.

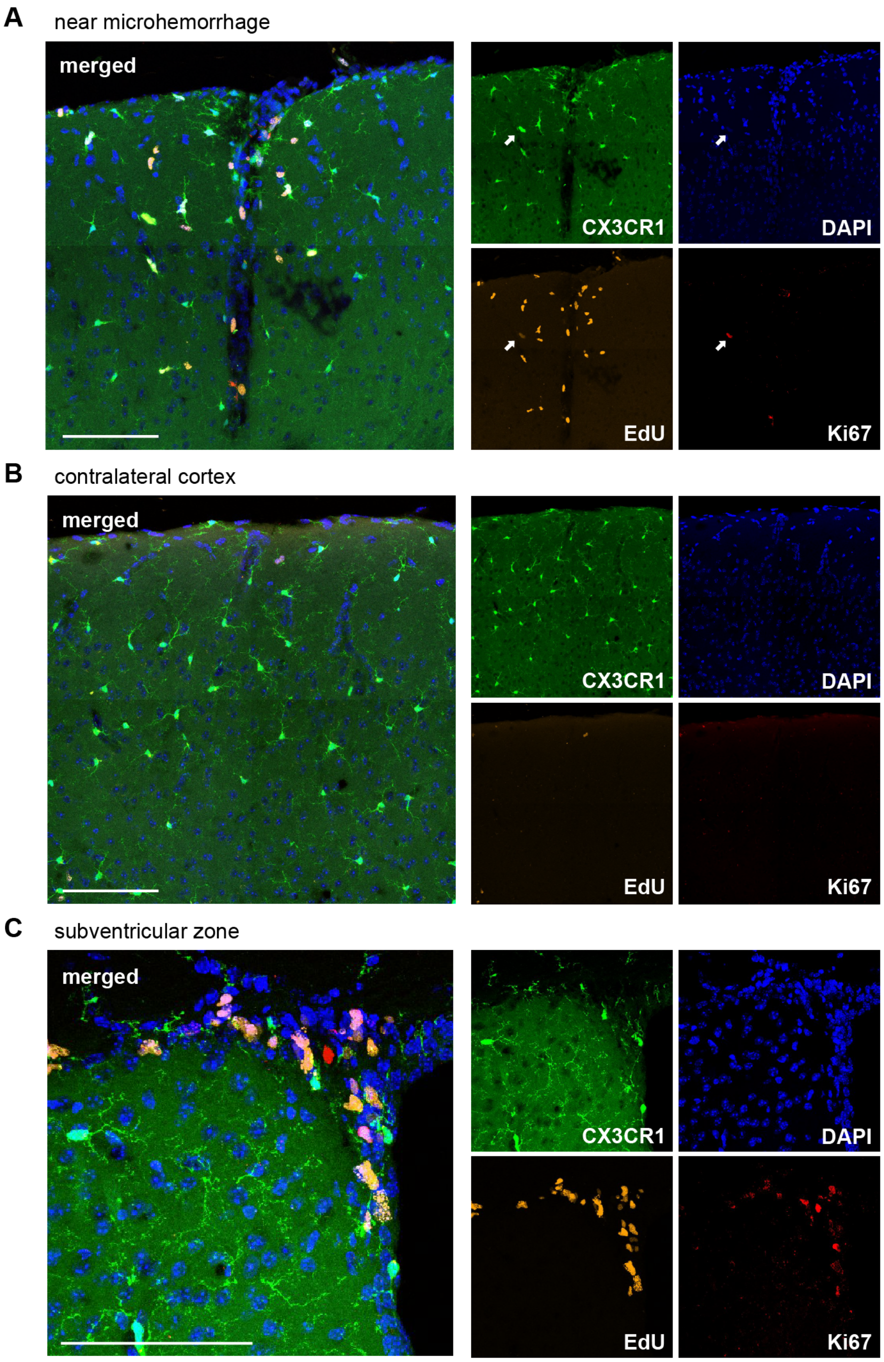

Supplementary figure III: Microglia proliferation was observed in the region surrounding a microhemorrhage. Histology images showing GFP-labeled microglia, DAPI labeled nuclei, EdU positive cells (EdU injected every eight hours for two days after microhemorrhage induction), and Ki67 expression (imunolabeled). (**A**) Section that intersects a microhemorrhage. The arrow indicates a cell that was labeled with EdU, Ki67, and GFP, indicating a microglia that was actively proliferating. (**B**) Negative control region from contralateral cortex. (**C**) Positive control region from sub-ventricular zone. Scale bars in A and B are 100 μm, in C it is 50 μm.

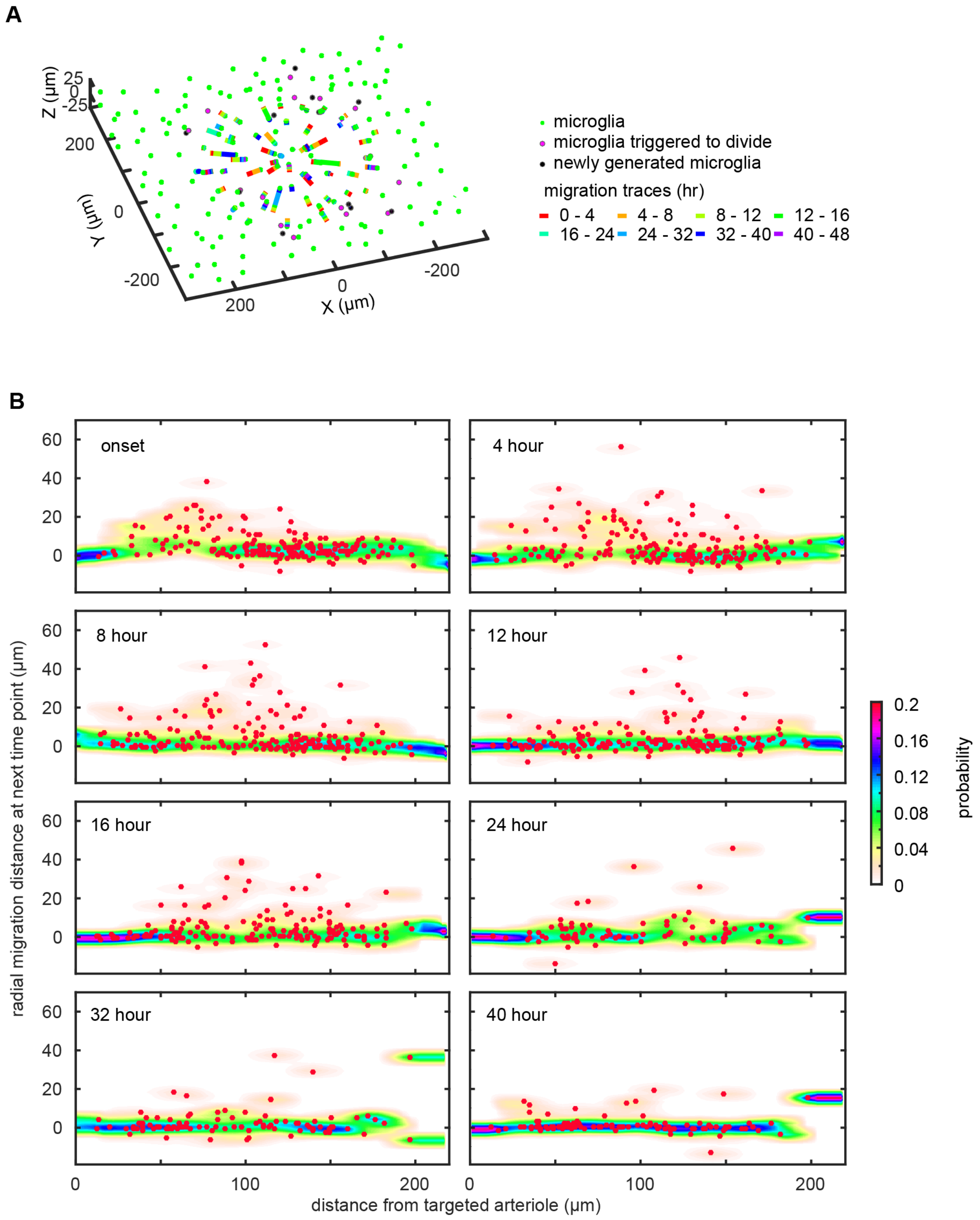

Supplementary figure IV: Microglia migration distance as a function of distance from the targeted arteriole and time after the injury. (**A**) Example of one simulation showing coordinates of microglia at two days after the microhemorrhage and the migration traces of individual microglia. Microglia committed to division and those newly generated are also indicated. (**B**) Red dots indicate experimental measurements of microglia migration from in vivo imaging (e.g. Fig. 3C and D). The average migration distance from this same data is shown in Fig. 3D. The color map shows the probability distribution of

migration distance for a microglia at a given distance from, and time after, the microhemorrhage. This probability distribution was derived from the experimental data by calculating the density of points over a sliding window that was 5 μm high (migration distance) and 20 μm wide (distance from targeted arteriole) and then smoothing. This probability function was then used for the simulations shown in Fig. 5.

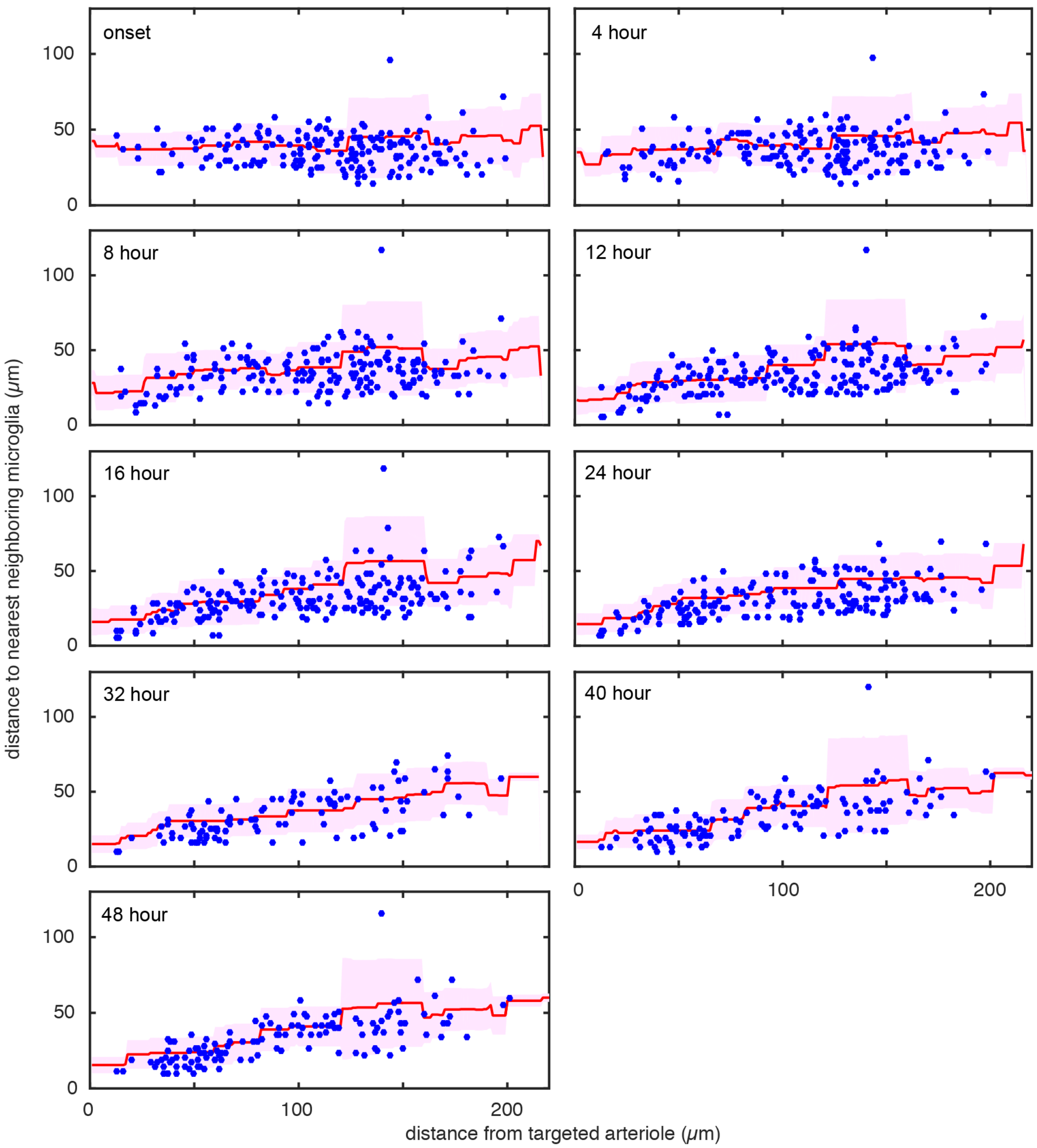

Supplementary figure V: Distance to nearest neighboring microglia as a function of distance from the targeted arteriole and time after the injury. Blue dots indicate experimental measurements of microglia nearest neighbor distances from the same data set used in Supplementary fig. 4 and Fig. 3D. Red line represents the mean, while the pink shadow represents ±1 SD from the mean.

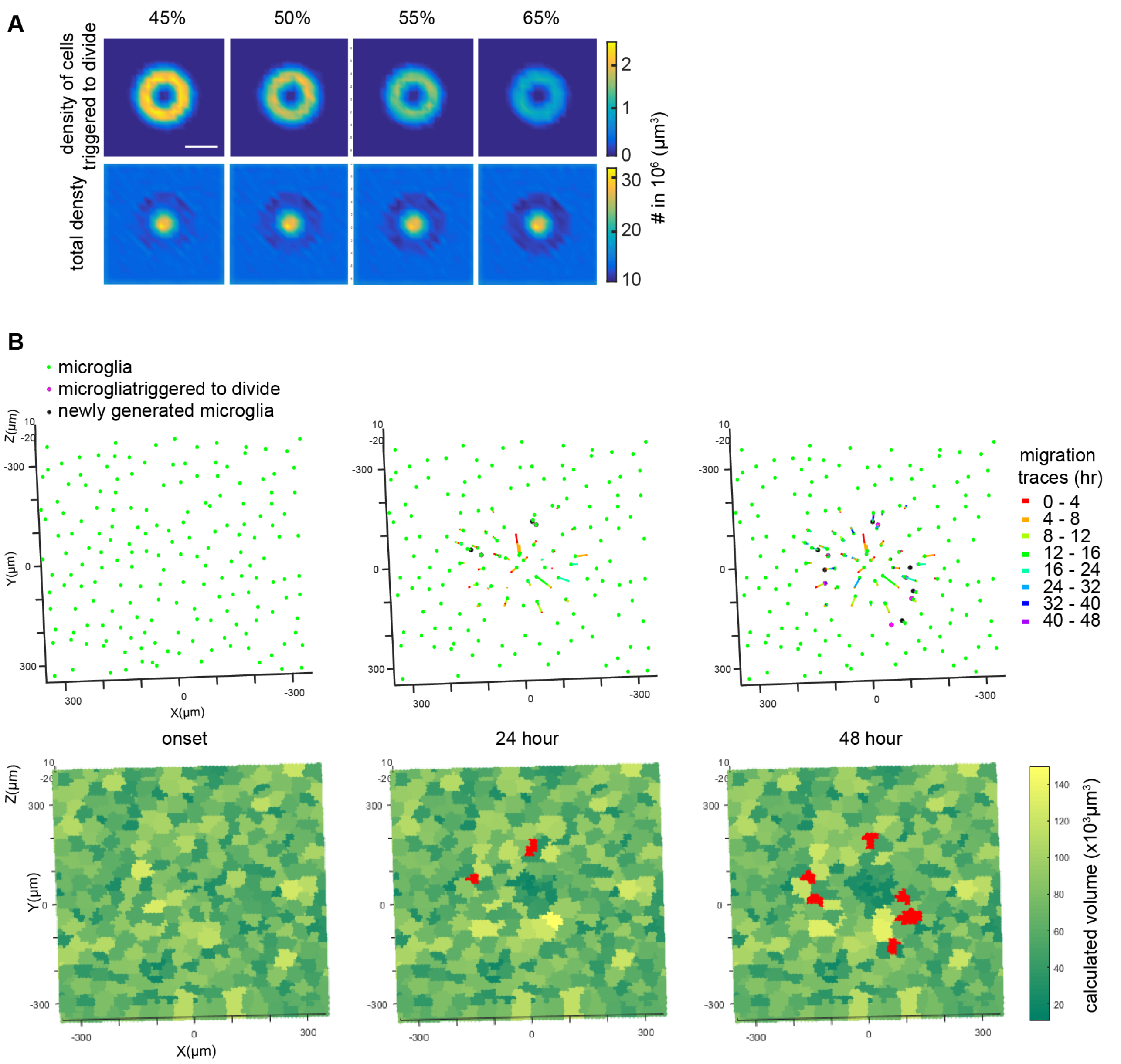

Supplementary figure VI: Calculation of the domain volume for each microglia using a topographic watershed algorithm over time after a microhemorrhage. When microglia migrated toward the injury, the domain volume of some microglia increased. (**A**) Map of the total density of microglia (top) and of the density of microglia committed to division (bottom) at 48 hrs after a microhemorrhage using models where microglia proliferation occurs when the domain volume of a microglia increases by more than the percentage indicated at the top of each column. (**B**) In this simulation, when the volume increased by more than 50% for a microglia, it committed to dividing and created a new microglia 8 hr later. Bottom panels show the microglia territories (note the image shows just a cross-sectional plane of these volumes) and the color indicates the size of the domain volume. Top panels show microglia locations and migration traces. The pink (black) circles indicate microglia that have committed to divide (are newly born). The red facets in the left panels also indicate these new microglia. The hemorrhage is at the center of the frame.

Supplemental References

1. Jung S, Aliberti J, Graemmel P, Sunshine MJ, Kreutzberg GW, Sher A, et al. Analysis of fractalkine receptor cx(3)cr1 function by targeted deletion and green fluorescent protein reporter gene insertion. *Molecular and cellular biology*. 2000;20:4106-4114
2. Schaefer BC, Schaefer ML, Kappler JW, Marrack P, Kedl RM. Observation of antigen-dependent cd8+ t-cell/ dendritic cell interactions in vivo. *Cellular immunology*. 2001;214:110-122
3. Saederup N, Cardona AE, Croft K, Mizutani M, Cotleur AC, Tsou CL, et al. Selective chemokine receptor usage by central nervous system myeloid cells in ccr2-red fluorescent protein knock-in mice. *PloS one*. 2010;5:e13693
4. Feng G, Mellor RH, Bernstein M, Keller-Peck C, Nguyen QT, Wallace M, et al. Imaging neuronal subsets in transgenic mice expressing multiple spectral variants of gfp. *Neuron*. 2000;28:41-51
5. Mildner A, Schmidt H, Nitsche M, Merkler D, Hanisch UK, Mack M, et al. Microglia in the adult brain arise from ly-6chiccr2+ monocytes only under defined host conditions. *Nat Neurosci*. 2007;10:1544-1553
6. Furuya T, Tanaka R, Urabe T, Hayakawa J, Migita M, Shimada T, et al. Establishment of modified chimeric mice using gfp bone marrow as a model for neurological disorders. *Neuroreport*. 2003;14:629-631
7. Holtmaat A, Bonhoeffer T, Chow DK, Chuckowree J, De Paola V, Hofer SB, et al. Long-term, high-resolution imaging in the mouse neocortex through a chronic cranial window. *Nature protocols*. 2009;4:1128-1144
8. Pologruto TA, Sabatini BL, Svoboda K. Scanimage: Flexible software for operating laser scanning microscopes. *BioMedical Engineering OnLine*. 2003;2:13
9. Cianchetti FA, Kim DH, Dimiduk S, Nishimura N, Schaffer CB. Stimulus-evoked calcium transients in somatosensory cortex are temporarily inhibited by a nearby microhemorrhage. *PloS one*. 2013;8: e65663
10. Nishimura N, Schaffer CB, Friedman B, Tsai PS, Lyden PD, Kleinfeld D. Targeted insult to subsurface cortical blood vessels using ultrashort laser pulses: Three models of stroke. *Nature Methods*. 2006;3:99-108
11. Rosidi NL, Zhou J, Pattanaik S, Wang P, Jin W, Brophy M, et al. Cortical microhemorrhages cause local inflammation but do not trigger widespread dendrite degeneration. *PloS one*. 2011;6:e26612
12. Schindelin J, Arganda-Carreras I, Frise E, Kaynig V, Longair M, Pietzsch T, et al. Fiji: An open-source platform for biological-image analysis. *Nat Methods*. 2012;9:676-682

Stroke Online Supplement

Table I. Checklist of Methodological and Reporting Aspects for Articles Submitted to *Stroke* Involving Preclinical Experimentation

Methodological and Reporting Aspects	Description of Procedures
Experimental groups and study timeline	☒ The experimental group(s) have been clearly defined in the article, including number of animals in each experimental arm of the study.<br>☒ An account of the control group is provided, and number of animals in the control group has been reported. If no controls were used, the rationale has been stated.<br>☒ An overall study timeline is provided.
Inclusion and exclusion criteria	☒ A priori inclusion and exclusion criteria for tested animals were defined and have been reported in the article.
Randomization	☒ Animals were randomly assigned to the experimental groups. If the work being submitted does not contain multiple experimental groups, or if random assignment was not used, adequate explanations have been provided.<br>☒ Type and methods of randomization have been described.<br>☒ Methods used for allocation concealment have been reported.
Blinding	☒ Blinding procedures have been described with regard to masking of group/treatment assignment from the experimenter. The rationale for nonblinding of the experimenter has been provided, if such was not feasible.<br>☒ Blinding procedures have been described with regard to masking of group assignment during outcome assessment.
Sample size and power calculations	☒ Formal sample size and power calculations were conducted based on a priori determined outcome(s) and treatment effect, and the data have been reported. A formal size assessment was not conducted and a rationale has been provided.
Data reporting and statistical methods	☒ Number of animals in each group: randomized, tested, lost to follow-up, or died have been reported. If the experimentation involves repeated measurements, the number of animals assessed at each time point is provided, for all experimental groups.<br>☒ Baseline data on assessed outcome(s) for all experimental groups have been reported.<br>☒ Details on important adverse events and death of animals during the course of experimentation have been provided, for all experimental arms.<br>☒ Statistical methods used have been reported.<br>☒ Numeric data on outcomes have been provided in text, or in a tabular format with the main article or as supplementary tables, in addition to the figures.
Experimental details, ethics, and funding statements	☒ Details on experimentation including stroke model, formulation and dosage of therapeutic agent, site and route of administration, use of anesthesia and analgesia, temperature control during experimentation, and postprocedural monitoring have been described.<br>☒ Different sex animals have been used. If not, the reason/justification is provided.<br>☒ Statements on approval by ethics boards and ethical conduct of studies have been provided.<br>☒ Statements on funding and conflicts of interests have been provided.
