# Supplementary figures and images for: Diverse Inflammatory Response After Cerebral Microbleeds Includes Coordinated Microglial Migration and Proliferation
Source: Stroke. 2018 May 29;49(7):1719–26. doi: 10.1161/STROKEAHA.117.020461 (PMC6019563; doi:10.1161/STROKEAHA.117.020461)

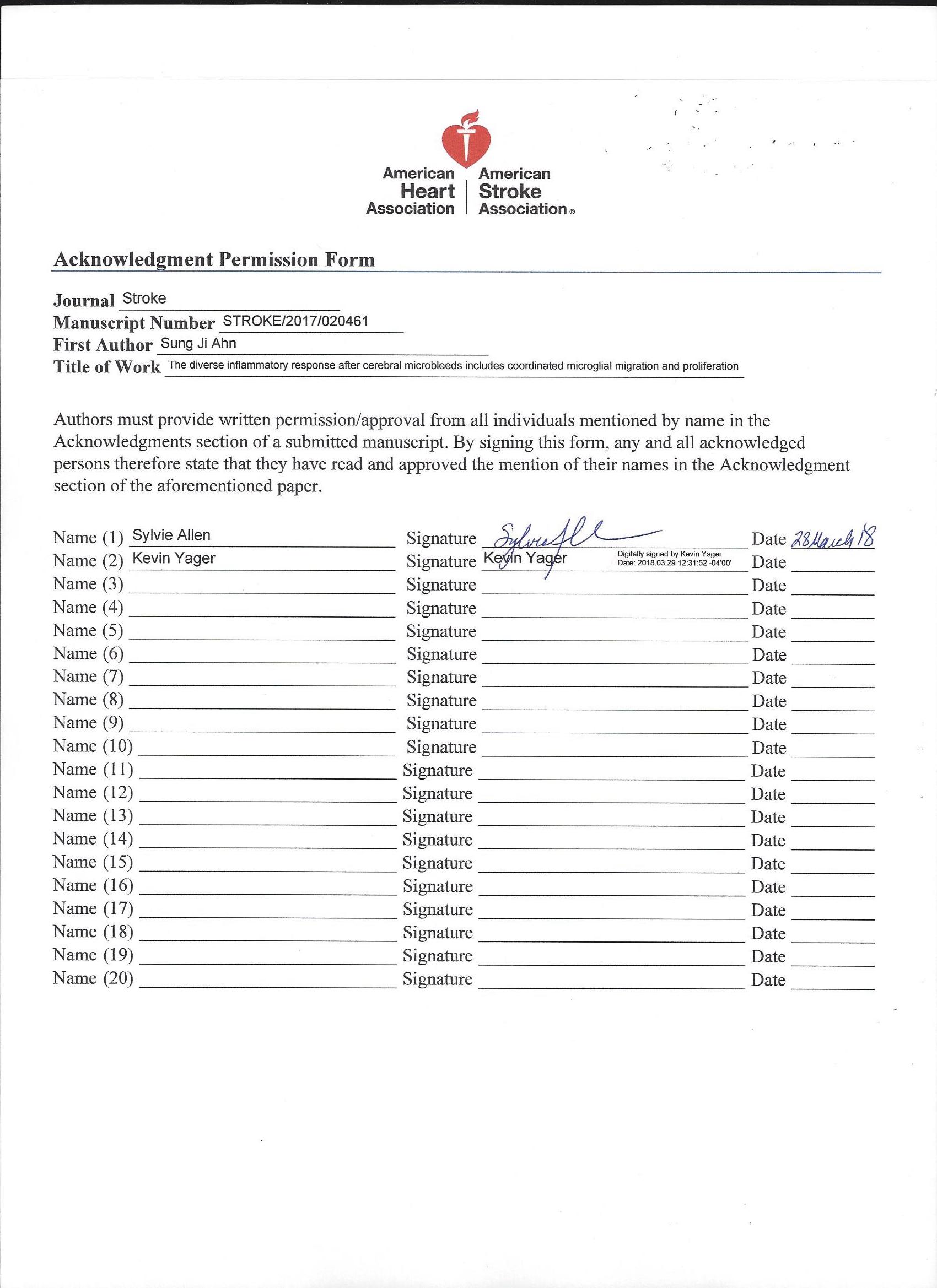

Supplement: Supplementary file 2 [file str-49-1719-s002.jpg]
