# Supplementary material for: Diverse Inflammatory Response After Cerebral Microbleeds Includes Coordinated Microglial Migration and Proliferation
Source: Stroke. 2018 May 29;49(7):1719–26. doi: 10.1161/STROKEAHA.117.020461 (PMC6019563; doi:10.1161/STROKEAHA.117.020461)
Supplement: Supplementary file 3 [file str-49-1719-s003.pdf]

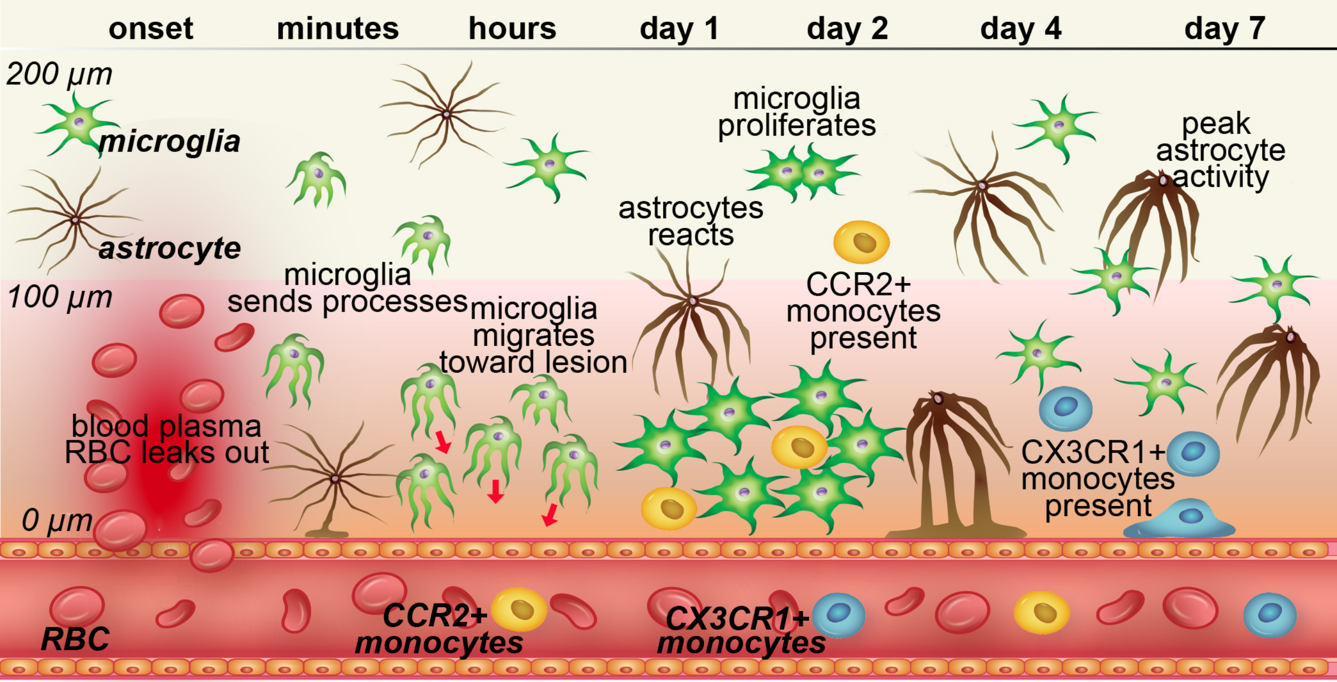

Using in vivo imaging approaches, we studied the response of some classes of inflammatory cells to a cortical microhemorrhage in mice. We found that brain microglia migrated toward the injury over a couple of days and then later proliferated in a shell surrounding the lesion, where microglia density had decreased due to that migration. A small number of blood-borne inflammatory cells were also found near the microhemorrhage. Astrocyte activation followed the microglia response, occurring in the same region where the microglia density increase was observed.
